# Evaluation of efficiency and effectiveness of different recruitment strategies for the FINGER‐NL multidomain lifestyle intervention trial via the Dutch Brain Research Registry

**DOI:** 10.1002/trc2.70017

**Published:** 2025-01-09

**Authors:** Lisa Waterink, Sietske A. M. Sikkes, Lion M. Soons, Sonja Beers, Yvonne Meijer‐Krommenhoek, Ondine van de Rest, Smidt Nynke, Joukje M. Oosterman, Erik Scherder, Kay Deckers, Yannick Vermeiren, Rianne A. A. de Heus, Sebastian Köhler, Wiesje M. van der Flier, MOCIA consortium, FINGER‐NL consortium, Marissa D. Zwan

**Affiliations:** ^1^ Alzheimer Center Amsterdam Neurology Vrije Universiteit Amsterdam, Amsterdam UMC VUmc Amsterdam The Netherlands; ^2^ Amsterdam Neuroscience, Neurodegeneration Amsterdam The Netherland; ^3^ Faculty of Behavioural and Movement Sciences Department of Clinical Neuro and Developmental Psychology Vrije Universiteit Amsterdam The Netherlands; ^4^ Alzheimer Centrum Limburg Department of Psychiatry and Neuropsychology School for Mental Health and Neuroscience (MHeNs) Maastricht University Maastricht The Netherlands; ^5^ Division of Human Nutrition and Health Wageningen University & Research Wageningen The Netherlands; ^6^ Department of Epidemiology University Medical Center Groningen (UMCG) University of Groningen (RUG) Groningen The Netherlands; ^7^ Donders Institute for Brain Cognition and Behaviour Radboud University Nijmegen The Netherlands; ^8^ Department of Geriatrics Department of Primary and Community Care Radboud University Medical Center Radboud University Medical Center Radboudumc Alzheimer Center Nijmegen The Netherlands; ^9^ Department of Epidemiology and Data Science Amsterdam University Medical Center Vrije Universiteit Amsterdam Amsterdam The Netherlands

**Keywords:** aging, dementia, lifestyle, recruitment, registries, trial

## Abstract

**INTRODUCTION:**

Recruitment of participants for intervention studies is challenging. We evaluated the effectiveness and efficiency of a participant recruitment campaign through an online registry for the FINGER‐NL study, a multi‐domain lifestyle intervention trial targeting cognitively healthy individuals aged 60–79 with dementia prevention potential. Additionally, we explored which recruitment strategy successfully reached individuals from underrepresented groups in research.

**METHODS:**

The campaign entailed seven recruitment strategies referring to The Dutch Brain Research Registry (DBRR): (1) Facebook advertisements, (2) appearance on national television, (3) newspaper articles, (4) researcher outreach, (5) patient organizations, (6) search engines, and (7) other. For each strategy, we describe the number of individuals (a) registered, (b) potentially eligible, and (c) included in FINGER‐NL. Subsequently, the efficiency, defined by the eligibility ratio (eligible/registered), and effectiveness, defined by the inclusion ratio (included/registered) were calculated. Associations between recruitment strategies and sociodemographic factors of underrepresented groups were tested with binomial logistic regressions.

**RESULTS:**

The campaign resulted in 13,795 new DBRR registrants, of which *n* = 3475 were eligible (eligibility ratio = 0.25) and *n* = 1008 were included (inclusion ratio = 0.07). The Facebook advertisements and television appearance resulted in the highest numbers of registrants (*n* = 4678 and *n* = 2182) which translated to the highest number of inclusions (*n* = 288 and *n* = 262). The appearance on national television (eligibility ratio = 0.35), newspaper articles (0.26), and Facebook campaigns (0.26) were the most efficient strategies. The national television appearance (inclusion ratio = 0.13) was the most effective strategy. The Facebook campaign and appearance on national television performed relatively better in recruiting individuals from underrepresented groups.

**DISCUSSION:**

A multipronged recruitment campaign via a national online recruitment registry is efficient and effective in recruiting and prescreening an adequate number of individuals aged 60–79 years with prevention potential for a multi‐site intervention trial within a limited time frame of 15 months. Social media advertisements and television are preferred strategies to recruit individuals from underrepresented groups.

**Highlights:**

- An online brain research registry recruited eligible participants successfully.
- Mass media recruitment strategies are efficient for reaching large numbers.
- Direct recruitment through researchers and patient organizations seems more effective.
- Online registries offer automated prescreening and alternatives for screen‐failures.
- Tailored strategies are needed to reach underrepresented groups to improve diversity.

## 1 INTRODUCTION

Recruiting participants for research and intervention studies is challenging and has been a major bottleneck for the progress in dementia research.^1^ Furthermore, screening for specific inclusion criteria demands significant staff efforts, often resulting in high screen failure rates, prolonged recruitment periods, and additional costs when delays occur.^1^ Currently, within dementia research, there is an increase in large lifestyle intervention trials to prevent or slow cognitive decline.^2^, ^3^, ^4^, ^5^ These trials target individuals with multiple risk factors for cognitive decline implying there is actual potential for preventive measures to be effective (i.e., prevention potential).^6^, ^7^ Finding participants with prevention potential from the general population requires creative recruitment strategies and an elaborate screening process.

The risk of dementia, or prevention potential, is unequally distributed across the population due to structural barriers and social determinants of health.^8^ For instance, individuals with lower socioeconomic status (SES) and from ethnic minorities face higher dementia risk^9^, ^10^, ^11^, ^12^, ^13^ which is partially explained by modifiable risk factors.^14^, ^15^, ^16^ Nonetheless, these groups are often underrepresented in dementia research,^17^, ^18^ possibly exacerbating disparities in dementia incidence and health outcomes^19^ as prevention trial results are not generalizable.^20^ Although recruitment strategies to include underrepresented groups have been developed and validated mainly in the United States,^21^ these strategies may not apply well to The Netherlands due to different ethnic composition and cultures. It is important to identify effective recruitment strategies for individuals less often participating in dementia research within The Netherlands.^22^

**RESEARCH IN CONTEXT**

1. **Systematic Review**: We reviewed the available literature through traditional sources (e.g., PubMed) on recruitment in dementia research. Recruitment challenges are widely acknowledged, and online recruitment registries are considered a promising recruitment approach. While some studies evaluate recruitment strategies, less is known about the recruitment of at‐risk individuals through an online registry.
2. **Interpretation**: The Dutch Brain Research Registry (DBRR), a nationwide online recruitment platform, employed an effective and efficient recruitment campaign by using multiple strategies, and recruited an adequate number of individuals (*n* = 1210) aged 60–79 years with prevention potential within 15 months. Results suggest that some strategies performed better in reaching individuals who are usually underrepresented in research. Online recruitment registries provide automated prescreening to support researchers and alternative participation options for prescreening failures.
3. **Future Directions**: Recruitment strategies of underrepresented groups need to be validated and adapted to The Netherlands' cultural diversity, to improve representation in the registry and ultimately in brain research and pharmacological trials nationwide.

In The Netherlands, the Dutch Brain Research Registry (DBRR) has been developed to support the recruitment and prescreening of research volunteers for neuroscience studies through a fully online registry.^23^ Other online registries worldwide have also shown to be successful in participant recruitment for trials in preclinical dementia and observational studies^24^, ^25^, ^26^, ^27^, ^28^, ^29^ illustrating that these platforms are promising tools to close the recruitment gap.^30^ Especially, since Internet use in The Netherlands is high among older adults (aged 65–75) compared to other European countries (80% vs. a European Union average of 40%),^31^ recruitment strategies referring to online registries may enable researchers to find eligible older participants more efficiently. While the challenges of recruitment are widely acknowledged,^32^ empirical data evaluating recruitment strategies through an online registry targeting older adults for prevention trials are lacking.

The multidomain lifestyle intervention trial FINGER‐NL (Box 1) is a randomized controlled trial targeting older adults at risk for cognitive decline, conducted in five sites throughout The Netherlands.^33^ Recruitment from the existing database of the DBRR and local cohorts was not sufficient to achieve the initial goal of 1206 randomized participants. For that reason, the DBRR supported the researchers with additional recruitment and launched a national recruitment campaign. This study evaluates the efficiency and effectiveness of different strategies as part of the recruitment campaign to recruit individuals with prevention potential, and eventually include participants for the FINGER‐NL trial. Recruitment for other multidomain lifestyle trials with similar targets took about 3 years.^34^, ^35^ We expect DBRR‐supported recruitment to accelerate trial inclusion, achieving target enrollment within 15 months to meet the project deadline. Outside the scope of this recruitment campaign, we explored which recruitment strategy reached underrepresented groups as guidance for future recruitment campaigns to increase diversity within the DBRR.

**BOX 1: FINGER‐NL Trial Design**

The FINGER‐NL trial is a two year, multicenter, randomized‐controlled trial on the effects of multidomain lifestyle changes on cognition ^33^. This trial targets adults aged between 60 and 79 years with increased risk of cognitive decline based on both modifiable risk factors and non‐modifiable risk factors. Individuals are eligible when they have two or more modifiable risk factors and at least one non‐modifiable risk factor. Modifiable risk factors include self‐reported hypertension, hypercholesterolemia, high body mass index (BMI), physical inactivity, unhealthy diet, and cognitive inactivity. Non‐modifiable risk factors were subjective memory complaints or a first‐degree relative with a diagnosis of dementia. Other inclusion criteria are adequate fluency in Dutch and internet access at home. Most important exclusion criteria of the study are diagnosis of mild cognitive impairment and dementia or substandard performance on the Telephone Interview for Cognitive Status test (TICS) ^36^. The FINGER‐NL trial is conducted at five sites throughout the Netherlands (Amsterdam, Maastricht, Wageningen, Groningen and Nijmegen) and registered on clinicaltrials.gov (NCT05256199).

## 2 METHODS

### 2.1 The DBRR: Recruitment and prescreening

**FIGURE 1:**
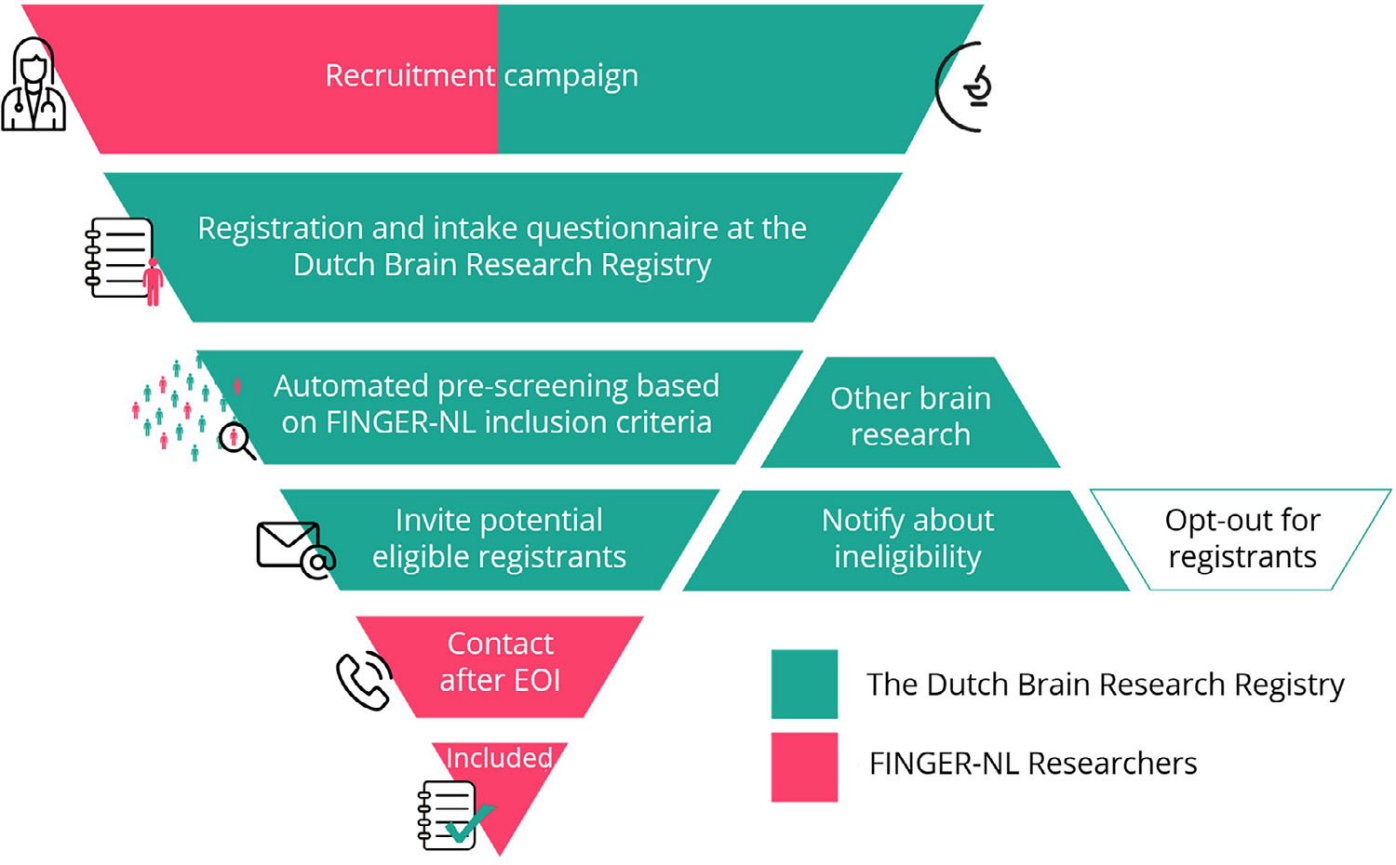
The recruitment flow of the DBRR in collaboration with the FINGER‐NL researchers. DBRR, Dutch Brain Research Registry; EOI, expression of interest.

The DBRR (in Dutch: Hersenonderzoek.nl) is a nationwide online registry for participant recruitment for brain disease studies in The Netherlands. Upon subscription, DBRR registrants fill out a basic questionnaire about personal‐, health‐, and lifestyle information. Based on this information and study‐specific inclusion criteria, registrants are automatically prescreened for eligibility by the DBRR and invited to participate in brain research. If potentially eligible, registrants receive a nonbinding study invitation per email asking about interest in participating. In this email, more information is provided, and registrants can express interest (or not) in study participation by a response button. If interested, researchers contact potential eligible registrants for further screening by telephone and to check study‐specific inclusion and exclusion criteria (Figure 1).

Before the recruitment campaign, the DBRR contained 21,700 registrants aged 18 years or older. Based on the inclusion and exclusion criteria of the FINGER‐NL trial (Box 1) and vicinity (postal code) to one of the five research sites, 1787 were eligible and received an invitation. Subsequently, 344 expressed their interest in participating in FINGER‐NL, and after telephone screening by the researchers, a total of 153 DBRR registrants were invited for a baseline visit. To reach the goal of 1206 randomized participants, an additional recruitment campaign was set up. The DBRR performed automated prescreening, and ineligible new registrants were promptly notified via email about their non‐qualification for the trial. The DBRR records the number of initially included registrants within FINGER‐NL and has no (complete) information on final randomization, screen failures, or drop‐outs as these are part of the trial. Here, we only report on participant recruitment through the DBRR.

### 2.2 Recruitment campaign for FINGER‐NL

The recruitment campaign consisted of numerous recruitment activities in close collaboration with the researchers. Recruitment activities were categorized into seven strategies: (1) Facebook advertisements; (2) the study launch on national television; (3) newspaper and online articles; (4) outreach from patient organizations; (5) search engine (e.g., Google); (6) other researcher outreach; and (7) other, not specified/specific (i.e., referrals via family, friends, and colleagues or recruitment activities that were not specific for FINGER‐NL). Recruitment activities were applied serially and adaptively, as based on the available number of potentially eligible participants, participating research sites were encouraged to perform additional recruitment activities referring participants to sign up at the DBRR. The recruitment campaign started in January 2022 and finished when the required number of participants was randomized. Table S1 contains a list of the recruitment activities per month grouped per strategy.

Upon subscription at DBRR, registrants reported their referral source. In addition, some online activities were directly traceable for each registrant with Urchin Tracking Module (UTM) tags attached to the hyperlinks referring to the DBRR website. Self‐reported referral source and UTM were manually cross‐checked (in cases of inconsistency, UTM codes were leading). Referral sources under the category “other”, were crosschecked by searching on keywords (e.g., “Facebook”, “news”), and subscription dates and dates of recruitment activities, and manually categorized in the appropriate referral source. In the following section, the seven recruitment strategies are described in more detail.

1. **Facebook advertisements**

Facebook advertisements were specifically designed and deployed by a professional media company (Figure S1) to target older adults aged 60–79 years who live near one of the research sites. Advertisements were designed to spark interest in the FINGER‐NL trial with lifestyle, brain health, and dementia‐related content by using different forms (i.e., video, quiz, and blog posts) which included a well‐known Dutch ambassador for brain health (E.S.; Figure S1). The advertisements ran from January 2022 until June 2022 and were relaunched from December 2022 till January 2023. Each specific advertisement was traceable with a unique UTM code. Advertisements referred to specifically designed landing pages related to the topic of interest on the DBRR website and contained more information about the relation between lifestyle and brain health, and research participation.

2. **Study launch on national television**

On 27‐01‐2022, the same ambassador (E.S.) together with one of the principal investigators of FINGER‐NL (W.M.F.) appeared on national television to launch the start of FINGER‐NL and with a call to participate, as part of a daily television show targeting older adults. Additionally, the broadcast network (https://www.omroepmax.nl/) included an article on its website and newsletter to maximize exposure. In the following year, the same network posted a short article on the experience of one of the first participants on their website, which was included in a second newsletter of the broadcast network to regain attention to the trial.

3. **Newspaper and online articles**

The sites at Maastricht, Wageningen, Nijmegen, and Groningen contacted newspapers around July 2022, December 2022, February 2023, and March 2023. Several (front‐page) articles were printed in national or regional newspapers and featured online. Some newspaper articles also included our ambassador E.S. To maximize exposure, articles were further distributed online by the researchers via social media.

4. **Outreach from patient organizations**

Dutch patient organizations *Alzheimer Netherlands* and the *Brain Foundation* were involved in the recruitment for FINGER‐NL. In Augustus 2022, *Alzheimer Netherlands* posted an online article on their website with a call to participate. In November 2022, the *Brain Foundation* made a call to participate in FINGER‐NL in their monthly newsletter.

5. **Search engine (e.g., Google)**

The DBRR has ongoing passive recruitment via search engine optimization. The DBRR homepage visibly featured lifestyle themed articles and the FINGER‐NL trial on the homepage to spark interest in participation.

6. **Other researcher outreach**

Throughout the year, researchers posted on social media platforms LinkedIn and Instagram for local recruitment and exposure. Flyers and posters were designed by a professional designer (Figure S2) and locally distributed at general practices, libraries, other community buildings, or in the neighborhoods surrounding the research site. Other researcher outreaches consisted of (online) presentations and workshops given at universities, Alzheimer café, community events, podcasts, or radio broadcasts (Table S1). In mid‐September 2022, the DBRR team and the researchers attended a national elderly exhibit aimed at people ages 50 years and older (https://www.50plusbeurs.nl/), which attracted 10,000–15,000 visitors a day. This event comprised a 5‐day booth from the DBBR including lifestyle‐themed presentations, a poster fair, and a meet‐up with researchers.

### 2.3 Measures

#### 2.3.1 Prevention potential

Prevention potential was based on self‐reported risk factors including modifiable and non‐modifiable risk factors for cognitive decline (based on the inclusion criteria for FINGER‐NL; Box 1).

#### 2.3.2 Sociodemographic and socioeconomic factors

As (socio)demographic and economic factors, we included age, sex, education, migration background, monthly household net income, and SES. Education was categorized into (1) vocational education or less (equivalent to ≤ 12 years of education) and (2) higher vocational or academic education (equivalent to ≥ 13 years of education). Monthly household net income was adjusted for household size by transforming it into a continuous equivalent income metric using the square root scale method by taking the midpoint of each category and dividing it by the square root of the number of individuals the income was intended to support.^36^ Equivalent monthly income was dichotomized as (1) < €2415 and (2) ≥ €2415 per month based on the median corrected monthly income of the Dutch population as reported by the Statistics Netherlands Bureau (CBS) in 2022 for ages 65–75.^37^ Individuals were classified as having a migration background when they or at least one of their parents was born outside of The Netherlands. Migration background was subdivided into Western (Europe, excluding Turkey; North America; Oceania; Indonesia; and Japan) and non‐Western (Africa; Latin‐Amerika; Asia, excluding Indonesia and Japan; Turkey).

### 2.4 Data and analysis

We describe the number of newly registered individuals at the DBRR, and, among these new registrants, identified the potentially eligible registrants, and registrants invited for the FINGER‐NL baseline visit, and subsequently calculated the efficiency and effectiveness of each recruitment strategy. Efficiency, operationalized as the eligibility ratio, was calculated by dividing the number of eligible individuals by the number of registered individuals (eligible/registered). Effectiveness, operationalized as the inclusion ratio, was calculated by dividing the number of included participants in FINGER‐NL by the number of registered individuals at DBRR (included/registered).

Subsequently, prevention potential, sociodemographic, and socioeconomic factors are described per recruitment strategy using descriptive statistics, and include absolute frequencies and percentages for categorical variables, and mean ± standard deviation or median and interquartile range where appropriate for continuous variables. Differences in sociodemographic and socioeconomic factors between recruitment strategies were tested with chi‐squared or Kruskal–Wallis test including post hoc tests corrected for multiple comparisons (*p *< 0.05).

Additionally, we explored which recruitment strategy performed best in recruiting individuals from underrepresented groups. Associations between recruitment strategy (predictor) and sociodemographic and socioeconomic factors of underrepresented groups (male sex, vocational education or less, migration background, and income below national median) were tested with binomial logistic regressions where the recruitment strategy “Other researcher outreach” was used as the reference group since it is the most traditional form of recruitment. The category “Other, not specified/specific” was excluded from the analysis since this did not involve active recruitment. For income “Prefer not to say” was imputed using a univariate imputation method with classification and regression trees prediction based on age, sex, and education since these missing were not random. Other observations with missing data were excluded from the models. Results of regression models were presented in odds ratios (OR) and their 95% confidence intervals (CI), where odds and a lower bound of 95%CI greater than 1 indicated a recruitment strategy in favor of underrepresented groups (*p* < 0.05). All statistical analyses were carried out in RStudio version 4.3.2.

## 3 RESULTS

### 3.1 Participant flow and differential effectiveness and efficiency

**FIGURE 2:**
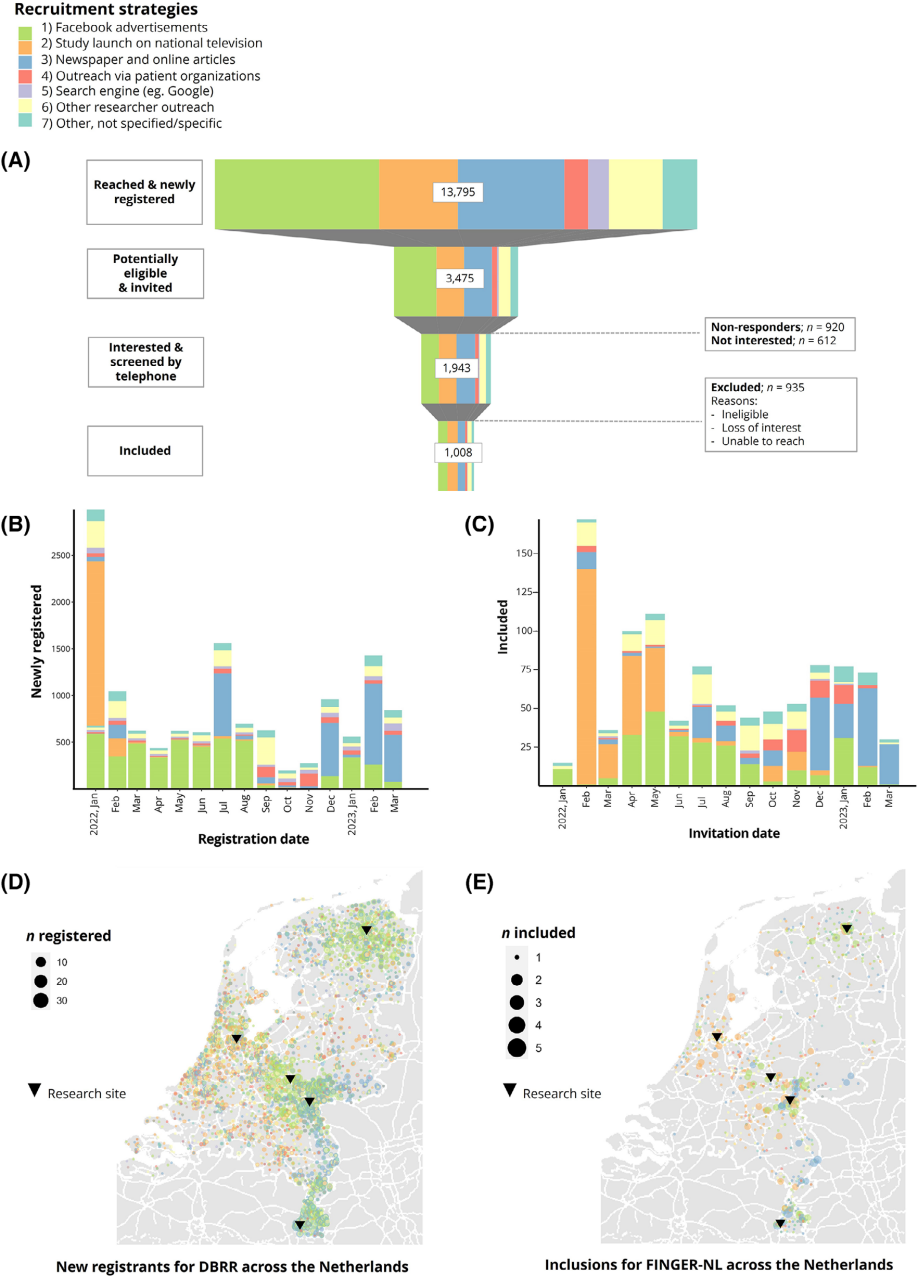
Results of national recruitment campaign via the DBRR in frequencies per strategy. (A) Overall recruitment flow colored by strategy. (B, C) Number of new and included registrants per strategy across months. Recruitment activities were performed adaptively based on the number of newly registrants to ensure sufficient inclusion across months. (D, E) Number of new and included registrants per strategy across The Netherlands. Recruitment was localized around study sites. Potentially eligible registrants were invited to nearest research site. *Research sites*: Groningen, Amsterdam, Wageningen, Nijmegen en Maastricht (from top to bottom and left to right). DBRR, Dutch Brain Research Registry.

The recruitment campaign ended in March 2023 (15 months in total; Figure 2B,C) as enough older adults with prevention potential had registered at the DBRR, and the randomization goal was expected to be reached soon hereafter. The recruitment campaign resulted in a total of 13,795 newly registered individuals at the DBRR across The Netherlands (Figure 2D), and 3475 registrants with prevention potential (eligibility ratio of 0.25; Table 1), of which 1008 were eventually invited for the FINGER‐NL baseline visit (inclusion ratio of 0.07; Figure 2,E). After the expression of interest, researchers contacted 1943 registrants for further telephonic screening after which 935 registrants were excluded due to ineligibility, loss of interest in the trial, or could not be reached (Figure 2A).

**TABLE 1:** Efficiency and effectiveness per recruitment strategy.

	Recruitment							
		Strategy (1)	Strategy (2)	Strategy (3)	Strategy (4)	Strategy (5)	Strategy (6)	Strategy (7)
Parameter	Overall	Facebook advertisements	Study launch on national television	Newspaper and online articles	Outreach via patient organizations	Search engine (e.g., Google)	Other researcher outreach	Other, not specified/specific
Total registered, *n (%)*	13,795 (100)	4678 (34)	2182 (16)	3001 (22)	709 (5)	618 (4)	1571 (11)	1036 (7)
Potentially eligible & invited, *n (%)*	3475 (100)	1192 (34)	761 (22)	776 (23)	149 (4)	47 (1)	333 (10)	217 (6)
Efficiency, eligibility ratio	0.25	0.26	0.35	0.26	0.21	0.08	0.21	0.21
Included, *n (%)*	1008 (100)	262 (26)	288 (29)	207 (21)	61 (6)	7 (1)	116 (12)	67 (7)
Effectiveness, inclusion ratio	0.07	0.06	0.13	0.07	0.09	0.01	0.07	0.06

*Note*: Results are presented in the number of participants and percentages of overall between brackets. Eligibility ratio = eligible/total registered. Inclusion ratio = included/total registered.

Comparing recruitment strategies, the highest numbers of new registrants were reached with Facebook advertisements (*n *= 4678, 34%), articles in newspapers (*n *= 3001, 22%), and national television appearances (*n *= 2182, 16%; Table 1). In particular, the Facebook advertisements with a lifestyle theme, and the well‐known ambassador outperformed all other Facebook advertisements (i.e., those with a brain health or dementia theme) in the number of registrations (*n/N* = 3216/4678, 69%; Table S2). The eligibility ratio varied across strategies between 0.08 and 0.35, with an appearance on national television (0.35), newspaper articles (0.26), and Facebook campaign (0.26) being the most efficient. These efficient strategies are all mass‐media exposures and mainly involved our well‐known ambassador and counted for 79% (*n *= 2.740) of the new registrants with prevention potential in total. The inclusion ratio varied between 0.01 and 0.13. The most effective strategy was the national television appearance (0.13) which allowed for more explanation about the target population and aim of the study.

### 3.2 Recruiting individuals from underrepresented groups

Newly registered individuals had a mean age of 66 years (interquartile range [IQR] = 62, 71), 33% were male (*n = *4487), 35% had a vocational education or less (*n *= 4793), and 11% had a migration background (*n *= 1444) of which only 190 (1% of total) were from non‐Western countries. 45% reported an equivalent monthly income below the national median; however, 18% preferred not to disclose their income (*n *= 2444). Regarding prevention potential, the national television appearance recruited especially more individuals with modifiable risk factors, and patient organizations recruited more individuals with non‐modifiable risk factors compared to other strategies (Table 2).

**TABLE 2:** Demographics, socioeconomic, and prevention potential factors of the newly registered sample (overall) and divided by recruitment strategy.

	Recruitment							
		Strategy (1)	Strategy (2)	Strategy (3)	Strategy (4)	Strategy (5)	Strategy (6)	
Parameter	Overall (*n* = 13,795)	Facebook advertisements (*n* = 4678)	Study launch on national television (*n* = 2182)	Newspaper and online articles (*n* = 3001)	Outreach via patient organizations (*n* = 709)	Search engine (e.g., Google) (*n* = 618)	Other researcher outreach (*n* = 1571)	*p*‐Value
Age in years (median [IQR])	66 [62, 71]	66^a^ [62, 70]	69^a^ [65, 73]	68^a^ [64, 73]	64^b^ [59, 70]	51^a^ [34, 64]	66^c^ [59, 71]	**<0.001**
**Sex, % (*n*)**								
Male	33 (4487)	24 (1115)^▼^	38 (821)^▲^	42 (1262)^▲^	24 (173)^▼^	30 (186)	34 (537)	**<0.001**
Female	68 (9295)	76 (3563)^▲^	62 (1,360)^▼^	58 (1737)^▼^	76 (535)^▲^	70 (429)	65 (1032)	
Other^*^	< 1 (13)	0 (0)	0 (1)	<1 (2)	<1 (1)	1 (3)	<1 (2)	
**Migration background, % (*n*)**								
Dutch ethnicity	85 (11727)	81 (3801)	89 (1939)	91 (2743)^▲^	85 (604)	72 (427)^▼^	85 (1337)	**<0.001**
Migration background	11 (1444)	9 (436)	11 (243)	9 (256)^▼^	11 (78)	20 (125)^▲^	11 (172)	
*Don't know/prefer not to say/missing* ^*^	5 (624)	9 (441)	0 (0)	<1 (2)	4 (27)	7 (46)	4 (62)	
**Education, % (*n*)**								
Vocational or less (0–12 years)	35 (4793)	42 (1973)^▲^	41 (883)^▲^	26 (789)^▼^	29 (202)^▼^	31 (191)	31 (492)^▼^	**<0.001**
Higher vocational or academic (≥ 13 years)	65 (9001)	58 (2705)^▼^	60 (1299)^▼^	74 (2212)^▲^	72 (507)^▲^	69 (427)	69 (1079)^▲^	
**Equivalent month income, % (*n*)**								
<€2415	45 (6222)	45 (2091)^▲^	56 (1228)^▲^	40 (1197)^▼^	42 (296)	49 (300)^▲^	44 (691)	**< 0.001**
≥€2415	37 (5129)	29 (1366)^▼^	33 (712)^▼^	50 (1495)^▲^	40 (281)	30 (183)^▼^	38 (607)	
*Don't know/prefer not to say/missing* ^*^	18 (2444)	25 (1171)	11 (242)	10 (310)	19 (132)	22 (135)	17 (237)	
**Prevention potential, modifiable risk factors**								
High BMI, % (*n*)	44 (6033)	50 (2325)^▲^	51 (1114)^▲^	34 (1018)^▼^	39 (277)	40 (246)	41 (651)	**<0.001**
Physical inactivity, % (*n*)	31 (4291)	30 (1375)^▼^	41 (890)^▲^	28 (838)^▼^	29 (203)	31 (192)	30 (466)	**<0.001**
Non‐Mediterranean diet, % (*n*)	32 (4361)	32 (1474)	33 (720)	27 (821)^▼^	32 (226)	44 (271)^▲^	33 (513)	**<0.001**
**Hypertension, % (*n*)**	30 (4173)	29 (1371)	41 (891)	30 (892)^▲^	28 (196)	17 (107)^▼^	28 (433)	**<0.001**
Hypercholesterolemia, % (*n*)	24 (3333)	22 (1044)^▼^	33 (717)^▲^	24 (723)	25 (178)	13 (83)^▼^	23 (355)	**<0.001**
Cognitively inactive, % (*n*)	18 (2538)	18 (845)	23 (504)^▲^	16 (478)^▼^	17 (118)	24 (146)^▲^	16 (254)	**<0.001**
**Prevention potential, non‐modifiable risk factors**								
Family member with dementia, % (*n*)	38 (5206)	34 (1592)^▼^	41 (886)^▲^	45 (1333)^▲^	46 (327)^▲^	25 (153)^▼^	36 (556)	**<0.001**
*Don't know/missing* ^*^	7 (911)	266 (6)	8 (173)	6 (175)	5 (38)	10 (61)	8 (128)	
Subjective memory complaints, % (*n*)	34 (4715)	31 (1443)^▼^	36 (788)	34 (1017)	39 (277)^▲^	50 (310)^▲^	33 (517)	**<0.001**

*Note*: *“*Strategy (7) Other not specified/specific” was not reported in this table. Equivalent month income is adjusted for household‐size and categories were based on the median household income in The Netherlands, as reported by the Statistics Netherlands Bureau in 2021. Differences were tested using Chi‐squared or Kruskal–Wallis, and post hoc analysis where appropriate.

Abbreviations: BMI, Body‐mass Index; IQR, interquartile range.

* Excluded from chi‐squared and post hoc analysis. Post hoc analysis, after Bonferroni correction for multiple comparisons, showed significant difference with:

^a^ all other recruitment strategies;

^b^ all other recruitment strategies except 6;

^c^ all other recruitment strategies except 4;

^▲^ being more often recruited (positively associated);

^▼^ being less often recruited (negatively associated) with this strategy.

**FIGURE 3:**
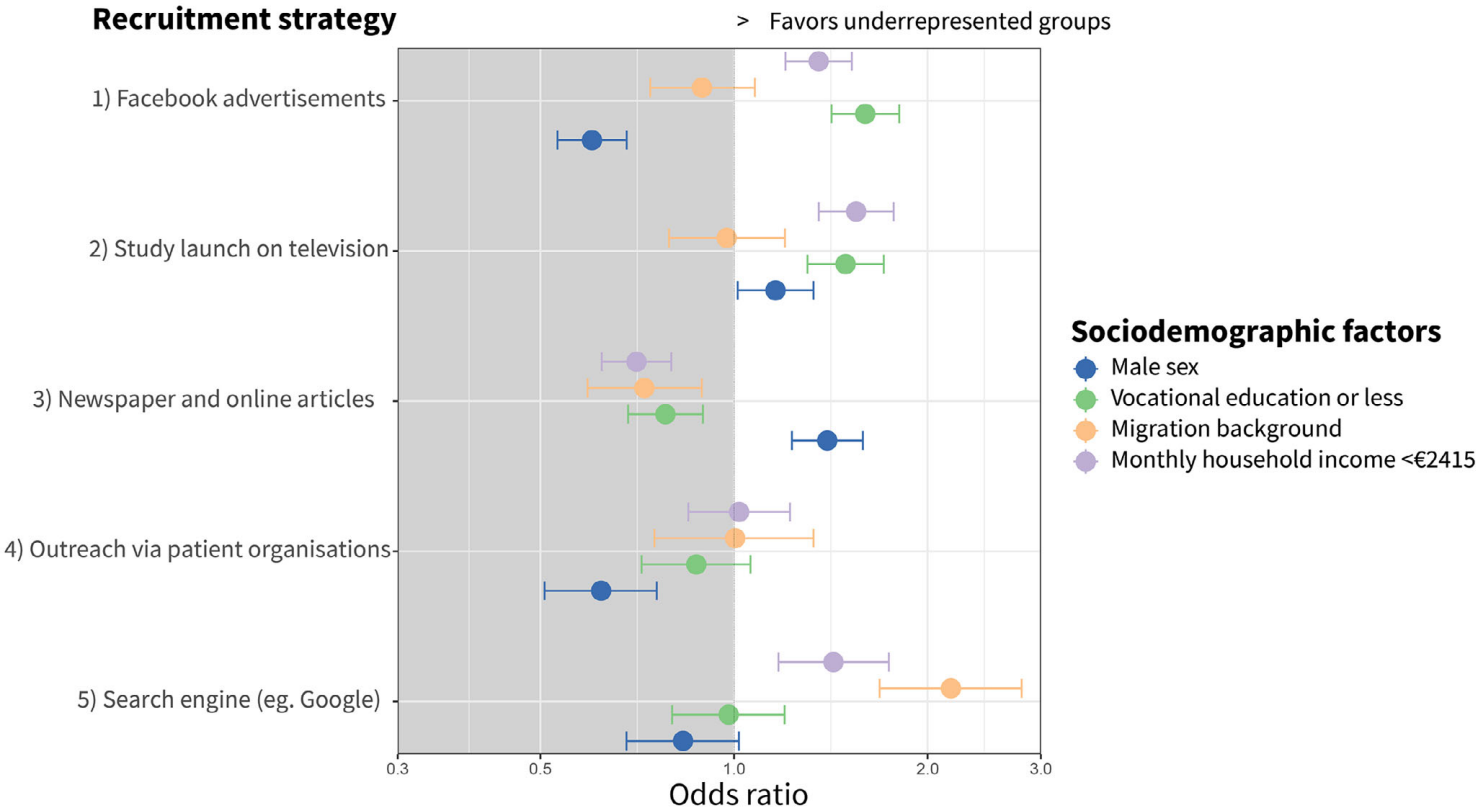
Recruitment strategies recruiting individuals from underrepresented groups to register at the DBRR. Forest plot displaying OR and 95% CI from unadjusted binomial regression models with "Strategy (6) Other researcher outreach" as the reference group. OR and lower bound of 95% CI greater than 1 display a significant association (*p* < 0.05) that favors the recruitment of underrepresented groups. CI, confidence interval; OR, odd ratio.

We found associations with some recruitment strategies and different targeted populations, in terms of (socio)demographic factors of underrepresented groups (Figure 3). Compared to the traditional recruitment strategy “Strategy 6: Other researcher outreach”, newspaper articles performed relatively better in recruiting males (OR_males_ 1.40; 95%CI: 1.23–1.59); however, less effective for all other underrepresented groups (Table S3). People with a migration background were hard to reach, yet these individuals best found the DBRR through the use of online search engines (OR_migration background_ 2.17; 95%CI: 1.68–2.09). The Facebook campaign (OR_vocational education_ 1.60: 95% CI: 1.42–1.81) and the appearance on national television (OR_vocational education_ 1.49: 95%CI: 1.30–1.71) recruited more individuals with a vocational education or less. The Facebook campaign (OR_<€2415_ 1.35: 95%CI: 1.20–1.52), appearance on national television (OR_<€2415_ 1.55: 95%CI: 1.35–1.71), and online search engines (OR_<€2451_ 1.42: 95%CI: 1.17–1.74) recruited more individuals with an income below the national median compared to individuals with higher income.

## 4 DISCUSSION

The recruitment campaign applying a variety of recruitment strategies and prescreening via the DBRR online recruitment registry was efficient and effective in including the required number (> 1000) of participants with prevention potential for the FINGER‐NL across five trial sites, within the limited timeframe of 15 months. For comparison, the recruitment periods from the original FINGER trial (*n* = 1260) using a population‐based cohort study, and Multidomain Alzheimer Preventive Trial (*n* = 1680) using various traditional strategies, were roughly 3 years.^34^, ^35^ Recruitment strategies including mass media such as tailored Facebook advertisements, national television appearances, and newspaper articles—especially when a well‐known ambassador for brain health was involved—were most efficient and responsible for 72% of new registrants for the DBRR with prevention potential. The television appearance by our ambassador and one of the Principal Investigators allowed for a more detailed explanation about the target population, resulting in relatively more inclusions for FINGER‐NL and proving the most effective. Our results provide relevant insights for the development of future recruitment campaigns for online registries.

Our campaign did not achieve a representative sample in terms of individuals with a migration background and lower SES compared to the Dutch population. Among individuals aged 65–80 in The Netherlands, 5% have a non‐Western migration background (primarily from Suriname, Morocco, and Turkey), whereas only 1% of our sample reflected this group. Similarly, approximately 70% of this age group have vocational education or less, yet only 35% were represented in our sample. This outcome was expected, as the campaign primarily targeted individuals with prevention potential and utilized non‐culturally tailored strategies. Nevertheless, our results show that certain recruitment strategies might be more effective in reaching individuals generally underrepresented in research. For instance, the Facebook advertisement and television appearance were more successful in reaching participants with vocational education or less (≤ 12 years of education) and a lower income, indicating their potential for recruiting individuals from lower socioeconomic backgrounds. In contrast, patient organizations and newspaper articles were less successful in reaching individuals from underrepresented groups but more effective in reaching those with non‐modifiable risk factors, such as having a family member with dementia or memory complaints. Notably, individuals with a migration background (i.e., they or at least one of their parents were born outside of The Netherlands) were more likely to register after independently searching for the DBRR online. The findings of this study, alongside current literature, offer valuable insights for designing more inclusive recruitment campaigns.^38^, ^39^, ^40^, ^41^, ^42^ For example, a culturally tailored recruitment campaign from the Brain Health Registry employing advertisements on social media efficiently and effectively increased the enrolment of Latino participants.^43^ Moving forward, the DBRR aims to enhance recruitment of underrepresented groups by validating existing strategies and adapting them to the cultural diversity of The Netherlands. This will improve representation within the registry and ultimately benefit brain research and pharmacological trials across the country.^44^

Previous studies on recruitment within the medical field of older adults reported high staff efforts and time investments.^45^, ^46^ For the DBRR, individuals register online, and the platform automatically prescreens large numbers of new registrants, and sends out programmed emails if individuals are (in)eligible accordingly. Without prescreening possibilities via the registry, on average, 14 persons needed to be reached and registered to include 1 participant. With the online registration and the automated prescreening process, researchers only needed to contact four potentially eligible participants to include one participant. This automated process significantly reduced the necessary phone calls for initial contact and prescreening for the research staff accelerating trial inclusion. This recruitment approach is also applicable for pharmacological trials, as has been shown by other online recruitment registries,^24^, ^47^ especially when combined with genetic screening.^47^, ^48^ To better facilitate demands of prevention trials—and given that a survey among registrants indicated a strong interest in participating in research on and disclosure of genetic risk for dementia^49^ ‐ the DBRR is currently conducting remote APOE‐ε4 screening to accelerate the inclusion of at‐risk individuals. Another advantage of recruitment through an online registry is that registrants can be prescreened for future brain research studies, utilizing their willingness and motivation to participate in research. The DBRR aims to keep registrants actively engaged and motivated for upcoming studies through newsletters and their website.

Strengths of this study include the use of various recruitment strategies and the ability to monitor their efficiency and effectiveness through the DBRR. Unfortunately, not all referral sources were directly traceable with UTM codes, properly self‐reported, or provided the exact number of individuals reached, and categorizing recruitment activities (e.g., “Other researchers outreach”) hinders direct comparison of specific activities. Additionally, large numbers of reached individuals did not complete the full registration process of the DBRR. The total number of registered individuals might therefore not present the actual number reached per recruitment strategy. For future campaigns, the DBRR will optimize the traceability of recruitment activities and the registration process. Moreover, a proportion of newly registered individuals did not respond to the initial invitation (*n* = 920; 26%) or were not interested (*n* = 612; 18%). Insight into reasons why people do not respond or are not interested in participation, and if this differs across strategies, could further improve the efficiency of DBRR recruitment campaigns. Moreover, while digital recruitment via an online registry can enhance a study's reach and accelerate trial inclusion, it may introduce selection bias due to varying levels of digital literacy or access. However, this concern was less relevant for FINGER‐NL, as Internet access was an inclusion criterion. Lastly, the categorization of migration background (i.e., the registrant or at least one parent born outside The Netherlands) has limitations due to cultural heterogeneity within the group and people who are born in the same country might have a different ethnic background. Unfortunately, low frequencies by country of birth precluded more detailed analyses.

## 5 CONCLUSION

To conclude, this study describes effective and efficient recruitment strategies for a multidomain lifestyle intervention trial to prevent cognitive decline. Our study underscores the need for a multipronged approach involving (mass) media—preferably including a well‐known brain health ambassador—collaboration with partners, and extensive outreach by researchers to recruit a sufficient number of older adults at risk for cognitive decline. Although we found some initial evidence of strategies more successful in reaching diverse populations, we recommend tailored strategies for underrepresented groups such as people with fewer years of education, lower SES, and migration background.

## CONFLICT OF INTEREST STATEMENT

S.A.M.S. provided consultancy services to Prothena Biosciences, Aribio, and Biogen, and she is part of the Scientific Advisory Board of Cogstate. All funds are paid to the institution. W.M.F. has performed contract research for Biogen MA Inc, and Boehringer Ingelheim. W.M.F. has been an invited speaker at Biogen MAInc, Danone, Eisai, Novonordisk, Web MD Neurology (Medscape), Springer Healthcare, European Brain Council. W.M.F. is consultant to Oxford Health Policy Forum CIC, Roche, Eisai, and Biogen MA Inc. W.M.F. participated on advisory boards of Biogen MAI inc, Roche, and EliLilly. All funding is paid to her institution. W.M.F. is a member of the steering committee of PAVE, and Think Brain Health. W.M.F. was associate editor of Alzheimer, Research & Therapy in 2020/2021. W.M.F. is associate editor at Brain. M.D.Z. is site coordinator of the phase 1/2 ASPIRE‐FTD clinical trial (NCT06064890) sponsored by AviadoBio. L.W., L.M.S., S.B., Y.M., O.R., N.S., J.M.O., E.S., K.D., Y.V.,R.A.A.H., and S.K. report no conflicts of interest. Author disclosures are available in the Supporting Information.

## ETHICS STATEMENT

The study was approved by the local Medical Ethical Committee. All registrants provided online informed consent for their data to be used for research purposes.

## Supporting information



Supporting Information

Supporting Information

## Data Availability

The datasets generated and/or analyzed during the current study are not publicly available due to ethical reasons since participants did not provide consent for data sharing.

## References

[1] Fargo KN , Carrillo MC , Weiner MW , Potter WZ , Khachaturian Z . The crisis in recruitment for clinical trials in Alzheimer's and dementia: an action plan for solutions. Alzheimers Dement. 2016;12(11):1113‐1115. 27836052 10.1016/j.jalz.2016.10.001

[2] Kivipelto M , Mangialasche F , Snyder HM , et al. World‐Wide FINGERS Network: a global approach to risk reduction and prevention of dementia. Alzheimers Dement. 2020;16(7):1078‐1094. 32627328 10.1002/alz.12123 PMC9527644

[3] Baumgart M , Snyder HM , Carrillo MC , Fazio S , Kim H , Johns H . Summary of the evidence on modifiable risk factors for cognitive decline and dementia: a population‐based perspective. Alzheimers Dement. 2015;11(6):718‐726. 26045020 10.1016/j.jalz.2015.05.016

[4] Ngandu T , Lehtisalo J , Solomon A , et al. A 2 year multidomain intervention of diet, exercise, cognitive training, and vascular risk monitoring versus control to prevent cognitive decline in at‐risk elderly people (FINGER): a randomised controlled trial. Lancet. 2015;385(9984):2255‐2263. 25771249 10.1016/S0140-6736(15)60461-5

[5] Coley N , Giulioli C , Aisen PS , Vellas B , Andrieu S . Randomised controlled trials for the prevention of cognitive decline or dementia: a systematic review. Ageing Res Rev. 2022;82:101777. 36336171 10.1016/j.arr.2022.101777

[6] Livingston G , Huntley J , Sommerlad A , et al. Dementia prevention, intervention, and care: 2020 report of the Lancet Commission. Lancet. 2020;396(10248):413‐446. 32738937 10.1016/S0140-6736(20)30367-6 PMC7392084

[7] Norton S , Matthews FE , Barnes DE , Yaffe K , Brayne C . Potential for primary prevention of Alzheimer's disease: an analysis of population‐based data. Lancet Neurol. 2014;13(8):788‐794. 25030513 10.1016/S1474-4422(14)70136-X

[8] Adkins‐Jackson PB , George KM , Besser LM , et al. The structural and social determinants of Alzheimer's disease related dementias. Alzheimers Dement. 2023;19(7):3171‐3185. 37074203 10.1002/alz.13027 PMC10599200

[9] Russ TC , Stamatakis E , Hamer M , Starr JM , Kivimäki M , Batty GD . Socioeconomic status as a risk factor for dementia death: individual participant meta‐analysis of 86 508 men and women from the UK. Br J Psychiatry. 2013;203(1):10‐17. 23818534 10.1192/bjp.bp.112.119479 PMC3696876

[10] Tang M‐X , Cross P , Andrews H , et al. Incidence of AD in African‐Americans, Caribbean Hispanics, and Caucasians in northern Manhattan. Neurology. 2001;56(1):49‐56. 11148235 10.1212/wnl.56.1.49

[11] Demirovic J , Prineas R , Loewenstein D , et al. Prevalence of dementia in three ethnic groups: the South Florida program on aging and health. Ann Epidemiol. 2003;13(6):472‐478. 12875807 10.1016/s1047-2797(02)00437-4

[12] Yaffe K , Falvey C , Harris TB , et al. Effect of socioeconomic disparities on incidence of dementia among biracial older adults: prospective study. BMJ. 2013;347:f7051. 24355614 10.1136/bmj.f7051 PMC3898154

[13] Cadar D , Lassale C , Davies H , Llewellyn DJ , Batty GD , Steptoe A . Individual and area‐based socioeconomic factors associated with dementia incidence in England: evidence from a 12‐year follow‐up in the English longitudinal study of ageing. JAMA Psychiatry. 2018;75(7):723‐732. 29799983 10.1001/jamapsychiatry.2018.1012 PMC6145673

[14] Deckers K , Cadar D , Van Boxtel MPJ , Verhey FRJ , Steptoe A , Köhler S . Modifiable risk factors explain socioeconomic inequalities in dementia risk: evidence from a population‐based prospective cohort study. J Alzheimers Dis. 2019;71(2):549‐557. 31424404 10.3233/JAD-190541 PMC6839472

[15] Dijkshoorn H , Diepenmaat ACM , Buster MCA , Uitenbroek D , Reijneveld SA . Sociaal‐economische status als verklaring van verschillen ingezondheid tussen Marokkanen en Nederlanders. Tijdschrift Voor Gezondheidswetenschappen. 2000;78:217‐222.

[16] Heger I , van Boxtel M , Deckers K , Bosma H , Verhey F , Köhler S . Socioeconomic position, modifiable dementia risk and cognitive decline: results of 12‐year Maastricht Aging Study. Int Psychogeriatr. 2024;36(7):574‐586. doi:10.1017/S1041610223000819 37905417

[17] Gilmore‐Bykovskyi AL , Jin Y , Gleason C , et al. Recruitment and retention of underrepresented populations in Alzheimer's disease research: a systematic review. Alzheimers Dement. 2019;5:751‐770. 10.1016/j.trci.2019.09.018 PMC6944728 31921966

[18] Elliott CL . Together we make the difference: National strategy for recruitment and participation in Alzheimer's and related dementias clinical research. Ethn Dis. 2020;30(Suppl 2):705‐708. 33250617 10.18865/ed.30.S2.705 PMC7683031

[19] Napoles AM , Chadiha LA . Advancing the science of recruitment and retention of ethnically diverse populations. Gerontologist. 2011;51(Suppl 1):S142‐S146. 21565815 10.1093/geront/gnr019 PMC3092974

[20] Van Der Flier WM , De Vugt ME , Smets EMA , Blom M , Teunissen CE . Towards a future where Alzheimer's disease pathology is stopped before the onset of dementia. Nature Aging. 2023;3(5):494‐505. 37202515 10.1038/s43587-023-00404-2

[21] Vila‐Castelar C , Fox‐Fuller JT , Guzmán‐Vélez E , Schoemaker D , Quiroz YT . A cultural approach to dementia—insights from US Latino and other minoritized groups. Nat Rev Neurol. 2022;18(5):307‐314. 35260817 10.1038/s41582-022-00630-z PMC9113534

[22] Grill JD , Sperling RA , Raman R . What should the goals be for diverse recruitment in Alzheimer clinical trials? JAMA Neurol. 2022;79(11):1097‐1098. 35969392 10.1001/jamaneurol.2022.2274

[23] Zwan MD , van der Flier WM , Cleutjens S , et al. Dutch Brain Research Registry for study participant recruitment: design and first results. Alzheimers Dement. 2021;7(1):e12132. 10.1002/trc2.12132 PMC7882519 33614897

[24] Weiner MW , Aaronson A , Eichenbaum J , et al. Brain health registry updates: an online longitudinal neuroscience platform. Alzheimers Dement. 2023;19(11):4935‐4951. 36965096 10.1002/alz.13077 PMC10518371

[25] Karagiannidou M , Stevens M , Knapp M , Cyhlarova E . Recruitment into dementia studies: experiences of researchers using the Join Dementia Research register. Int J Geriatr Psychiatry. 2022;37(1). doi:10.1002/gps.5629 34642964

[26] Jeon Y‐H , Shin M , Smith A , et al. Early implementation and evaluation of StepUp for dementia Research: an Australia‐wide dementia research participation and public engagement platform. Int J Environ Res Public Health. 2021;18(21):11353. 34769871 10.3390/ijerph182111353 PMC8583565

[27] Langbaum JB , High N , Nichols J , Kettenhoven C , Reiman EM , Tariot PN . The Alzheimer's Prevention Registry: a large internet‐based participant recruitment registry to accelerate referrals to Alzheimer's‐focused studies. J Prev Alzheimers Dis. 2020;7(4):242‐250. 32920626 10.14283/jpad.2020.31 PMC7534299

[28] Langbaum JB , Karlawish J , Roberts JS , et al. GeneMatch: a novel recruitment registry using at‐home APOE genotyping to enhance referrals to Alzheimer's prevention studies. Alzheimers Dement. 2019;15(4):515‐524. 30772251 10.1016/j.jalz.2018.12.007 PMC6461487

[29] Jimenez‐Maggiora GA , Bruschi S , Raman R , et al. TRC‐PAD: accelerating recruitment of AD clinical trials through innovative information technology. J Prev Alzheimers Dis. 2020;7(4):226‐233. 32920624 10.14283/jpad.2020.48 PMC7769128

[30] Grill JD . Recruiting to preclinical Alzheimer's disease clinical trials through registries. Alzheimers Dement. 2017;3(2):205‐212. 10.1016/j.trci.2017.02.004 PMC5399544 28439532

[31] Akkermans M . Internetgebruik ouderen fors toegenomen. Centraal Bureau voor de Statistiek (CBS); 2013.

[32] Grill JD , Karlawish J . Addressing the challenges to successful recruitment and retention in Alzheimer's disease clinical trials. Alzheimers Res Ther. 2010;2(6):34. 21172069 10.1186/alzrt58 PMC3031880

[33] Deckers K , Zwan MD , Soons LM , et al. A multidomain lifestyle intervention to maintain optimal cognitive functioning in Dutch older adults‐study design and baseline characteristics of the FINGER‐NL randomized controlled trial. Alzheimers Res Ther. 2024;16(1):126. 38872204 10.1186/s13195-024-01495-8 PMC11170777

[34] Ngandu T , Lehtisalo J , Levälahti E , et al. Recruitment and baseline characteristics of participants in the Finnish Geriatric Intervention Study to prevent cognitive impairment and disability (FINGER)—a randomized controlled lifestyle trial. Int J Environ Res Public Health. 2014;11(9):9345‐9360. 25211775 10.3390/ijerph110909345 PMC4199023

[35] Carrie I , Abellan Van Kan G , Gillette‐Guyonnet S , et al. Recruitment strategies for preventive trials. The MAPT study (Multidomain Alzheimer Preventive Trial). J Nutr Health Aging. 2012;16(4):355‐359.22499458 10.1007/s12603-012-0046-8

[36] OECD . Divided We Stand: Why Inequality Keeps Rising. OECD Publishing; 2011.

[37] StatisticsNetherlands . StatLine: Verdeling Gestandaardiseerd inkomen. Centraal Bureau voor de Statistiek (CBS); 2023

[38] Stuber JM , Middel CNH , Mackenbach JD , Beulens JWJ , Lakerveld J . Successfully recruiting adults with a low socioeconomic position into community‐based lifestyle programs: a qualitative study on expert opinions. Int J Environ Res Public Health. 2020;17(8):2764. 32316344 10.3390/ijerph17082764 PMC7215437

[39] Mindt MR , Okonkwo O , Weiner MW , et al. Improving generalizability and study design of Alzheimer's disease cohort studies in the United States by including under‐represented populations. Alzheimers Dement. 2023;19(4):1549‐1557. 36372959 10.1002/alz.12823 PMC10101866

[40] Weiner MW , Veitch DP , Miller MJ , et al. Increasing participant diversity in AD research: Plans for digital screening, blood testing, and a community‐engaged approach in the Alzheimer's Disease Neuroimaging Initiative 4. Alzheimers Dement. 2023;19(1):307‐317. 36209495 10.1002/alz.12797 PMC10042173

[41] Raman R , Quiroz YT , Langford O , et al. Disparities by race and ethnicity among adults recruited for a preclinical Alzheimer disease trial. JAMA Netw Open. 2021;4(7):e2114364. 34228129 10.1001/jamanetworkopen.2021.14364 PMC8261604

[42] Choi I , Milne DN , Glozier N , Peters D , Harvey SB , Calvo RA . Using different facebook advertisements to recruit men for an online mental health study: engagement and selection bias. Internet Interventions. 2017;8:27‐34. 30135825 10.1016/j.invent.2017.02.002 PMC6096306

[43] Ashford MT , Camacho MR , Jin C , et al. Digital culturally tailored marketing for enrolling Latino participants in a web‐based registry: baseline metrics from the brain health registry. Alzheimers Dement. 2023;19(5):1714‐1728. 36193827 10.1002/alz.12805 PMC10070578

[44] Greenberg BD , Lemere CA , Barnes LL , et al. Prescribing anti‐amyloid immunotherapies to treat Alzheimer's disease: fully informing patient decisions. Alzheimers Dement. 2023;9(4):e12426. 10.1002/trc2.12426 PMC10549961 37799322

[45] Greimel S , Wyman JF , Zhang L , Yu F . Recruitment and screening methods in Alzheimer's disease research: The FIT‐AD trial. J Gerontol A Biol Sci Med Sci. 2022;77(3):547‐553. 33780529 10.1093/gerona/glab092 PMC8893175

[46] Barreto PDS , Rolland Y , Cesari M , Dupuy C , Andrieu S , Vellas B . Effects of multidomain lifestyle intervention, omega‐3 supplementation or their combination on physical activity levels in older adults: secondary analysis of the Multidomain Alzheimer Preventive Trial (MAPT) randomised controlled trial. Age Ageing. 2017;47(2):281‐288. 10.1093/ageing/afx164 29136094

[47] Bardach SH , Langbaum JB , Kebodeaux CS , Jicha GA . Real‐world site experiences with GeneMatch: the role of a recruitment‐related registry in the context of local site effort to support Alzheimer disease prevention research. Alzheimer Dis Assoc Disord. 2021;35(2):148‐152. 33273159 10.1097/WAD.0000000000000425 PMC8137512

[48] Fockler J , Kwang W , Ashford MT , et al. Brain health registry GenePool study: a novel approach to online genetics research. Alzheimers Dement. 2021;7(1):e12118. 10.1002/trc2.12118 PMC7882536 33614891

[49] Waterink L , Masselink LA , Van Der Lee SJ , et al. Interest in genetic susceptibility testing and disclosure of AD dementia risk in cognitively normal adults: a survey study. Alzheimers Res Ther. 2024;16(1):1. 38167083 10.1186/s13195-023-01364-w PMC10759504

