# Erratum to “Evaluation of efficiency and effectiveness of different recruitment strategies for the FINGER‐NL multidomain lifestyle intervention trial via the Dutch Brain Research Registry”

**DOI:** 10.1002/trc2.70082

**Published:** 2025-06-05

Waterink L, Sikkes SAM, Soons LM, et al. Evaluation of efficiency and effectiveness of different recruitment strategies for the FINGER‐NL multidomain lifestyle intervention trial via the Dutch Brain Research Registry *Alzheimer's Dement*. 2025;11(1):e70017. doi: 10.1002/trc2.70017.

In the Author list, the first and last name of the seventh author are reversed. This should have been:

Nynke Smidt (https://orcid.org/0000‐0002‐2778‐8841)

We apologize for this error.

