# Supplementary material for: Evaluation of efficiency and effectiveness of different recruitment strategies for the FINGER‐NL multidomain lifestyle intervention trial via the Dutch Brain Research Registry
Source: Alzheimers Dement (N Y). 2025 Jan 9;11(1):e70017. doi: 10.1002/trc2.70017 (PMC11712179; doi:10.1002/trc2.70017)
Supplement: Supplementary file 1 — Supporting Information [file TRC2-11-e70017-s001.docx]

**
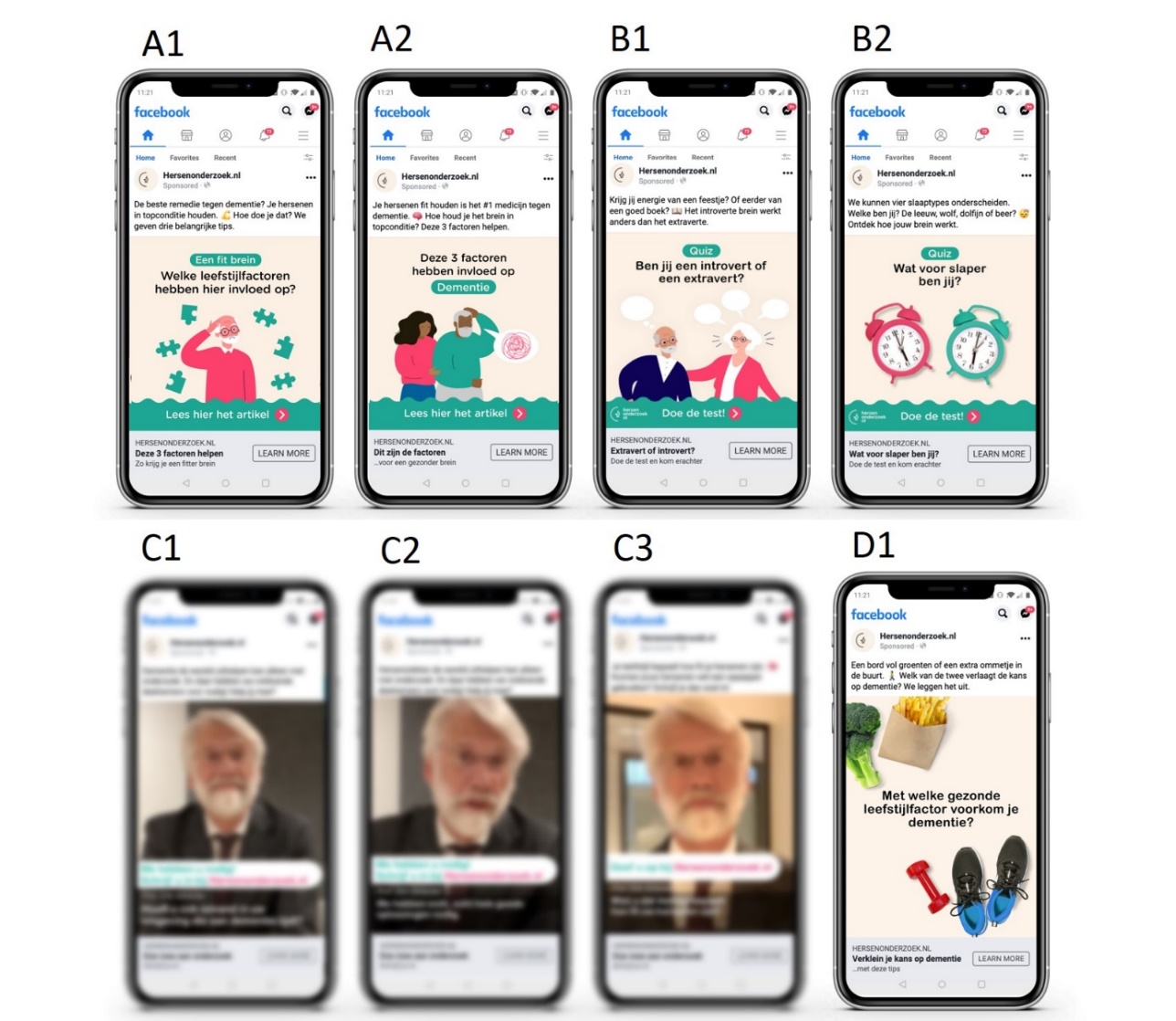
**

**Supplementary Figure 1.** Facebook advertisement developed by a content and marketing bureau (https://yune.nl/) for targeted recruitment of participants for the multi-domain lifestyle intervention trial FINGER-NL which were automatically optimized. A) blog-post with two themes A1 = brain health and A2 = dementia; B) lifestyle themed quizzes: B1 = social engagement and B2 = sleep; C) video ads which included a nationally well-known ambassador (E.S.) who advocates a healthy lifestyle to improve brain-health and varied in theme C1 = dementia; C2 = brain diseases and C3 = lifestyle; D1) animated picture prevention of dementia with blogpost.

**Supplementary Table 1.** Specific recruitment activities from January 2022 till March 2023 categorized by strategy.

| **Date** | **Strategy** | **Referral source** | **Regional/national** | **Specific recruitment activity** |
| --- | --- | --- | --- | --- |
| Jan-22 - Mar-23 | Facebook campaign | Facebook campaign | National | Tailored lifestyle campaign |
| Jan-22 | Study launch on national television ES &WF | Television | National | TV broadcast at elderly network with our ambassador (E.S.) and dementia researcher (W.F.) |
| Sep-22 | Other researcher outreach | (Online) event or lecture | National | Elderly exhibit five day booth including lifestyle themed presentations, a poster fair and meet-up with researchers |
| Aug-22 | Other researcher outreach | (Online) newsarticle | Regional - Maastricht | Daily newspaper in print |
| Sep-22 | Other researcher outreach | (Online) newsarticle | Regional - Maastricht | Daily newspaper in print |
| Jan-22 | Other researcher outreach | Social media other | National | LinkedIn - Study launch |
|  | Other researcher outreach | Flyers/posters | Regional – Amsterdam | ----- |
| Jul-22 | Other researcher outreach | Social media other | Regional – Maastricht | LinkedIn - Call for participants |
| Feb-22 | Other researcher outreach | Social media other | Regional - Maastricht | News item on website from university medical centre |
| Nov-22 | Other researcher outreach | (Online) newsarticle | Regional - Maastricht | News item in magazine from university medical centre |
|  | Other researcher outreach | Flyers/posters | Regional - Groningen | Flyers/posters distribution to general practitioner |
| Jul-22 | Other researcher outreach | Facebook | Regional - Wageningen | Local Facebook group |
| Jul-21 - Dec-22 | Other researcher outreach | Flyers/posters | Regional - Nijmegen | Distribution on site and to general practitioners/nursing homes/physiotherapists/community centers or community centers/libraries |
| Jan-23 | Other researcher outreach | Local cohort | Regional - Nijmegen | Local cohort different department Radboud university medical centre |
| Jan-22- Feb-23 | Other researcher outreach | Local cohort | Regional - Wageningen | Local cohort Wageningen University |
| Apr-22 - Feb-23 | Other researcher outreach | Flyers/posters | Regional - Wageningen | Flyers/posters distribution among pharmacies, physiotherapists, supermarkets, shops, senior complexes and museums |
| Oct-22 - Nov-22 | Other researcher outreach | Flyers/posters | Regional - Wageningen | Flyers/posters distribution of bookmarks as flyers at library and bookshops |
| Oct-22 | Outreach from patient organizations | (Patient) organization | National | Alzheimer Nederland - Online article including interview with FINGER-NL researcher (K.D.) |
| Nov-22 | Outreach from patient organizations | (Patient) organization | National | Hersenstichting – featured in online newsletter and improved visibility on website |
| Feb-23 | Other researcher outreach | (Online) newsarticle + social media other | National | Website for dietitian |
| Feb-23 | Other researcher outreach | (Online) newsarticle | National | Website about nutrition |
| Sep-21 | Other researcher outreach | (Online) newsarticle | National | News item about brain health |
| Jul-22 | Other researcher outreach | (Online) newsarticle | National | News items about dementia risk |
| Mar-23 | Other researcher outreach | (Online) newsarticle | Regional - Groningen | Daily newspaper |
| Feb-22 | Other researcher outreach | (Online) newsarticle | Regional - Maastricht | News article regional network |
| Aug-22 | Other researcher outreach | (Online) newsarticle | Regional - Maastricht | Interview with researcher in magazine |
| Feb-22 | Other researcher outreach | Television | Regional - Maastricht | News item regional television |
| Nov-22 | Other researcher outreach | (Patient) organization | Regional - Maastricht | Alzheimer Nederland - Presentation at Alzheimer Café |
| Mar-22 | Other researcher outreach | (Online) event or lecture | Regional – Maastricht | Workshop ‘’Keep your brain healthy! What can you do yourself?’’ |
| Apr-22 | Other researcher outreach | (Online) event or lecture | Regional – Maastricht | Workshop ‘’Keep your brain healthy! What can you do yourself?’’ |
| Sep-22 | Other researcher outreach | (Online) event or lecture | Regional – Maastricht | Workshop ‘’Keep your brain healthy! What can you do yourself?’’ |
| Oct-22 | Other researcher outreach | (Online) event or lecture | Regional – Maastricht | Lecture ‘’Prevention of dementia!?’’ |
| Nov-22 | Other researcher outreach | (Online) event or lecture | Regional – Maastricht | Lecturer ‘Dementia risk reduction’. Educational program for casemanagers dementia, |
| Dec-22 | Other researcher outreach | (Online) newsarticle | Regional – Maastricht | Daily newspaper online and in print |
| Dec-22 | Other researcher outreach | (Online) event or lecture | Regional – Maastricht | Night of Science |
| Jan-22 - Mar-23 | Other researcher outreach | (Online) newsarticle | Regional - Wageningen | Voedingsonderzoek WUR werving |
| Oct-22 | Other researcher outreach | (Online) newsarticle | Regional - Wageningen | Catholic Advocacy Organization for Seniors |
| Oct-22 | Other researcher outreach | (Online) newsarticle | Regional - Wageningen | Daily newspaper online and in print |
| Feb-23 | Other researcher outreach | (Online) newsarticle | Regional – Wageningen/Nijmegen | Daily newspaper online and in print |

**
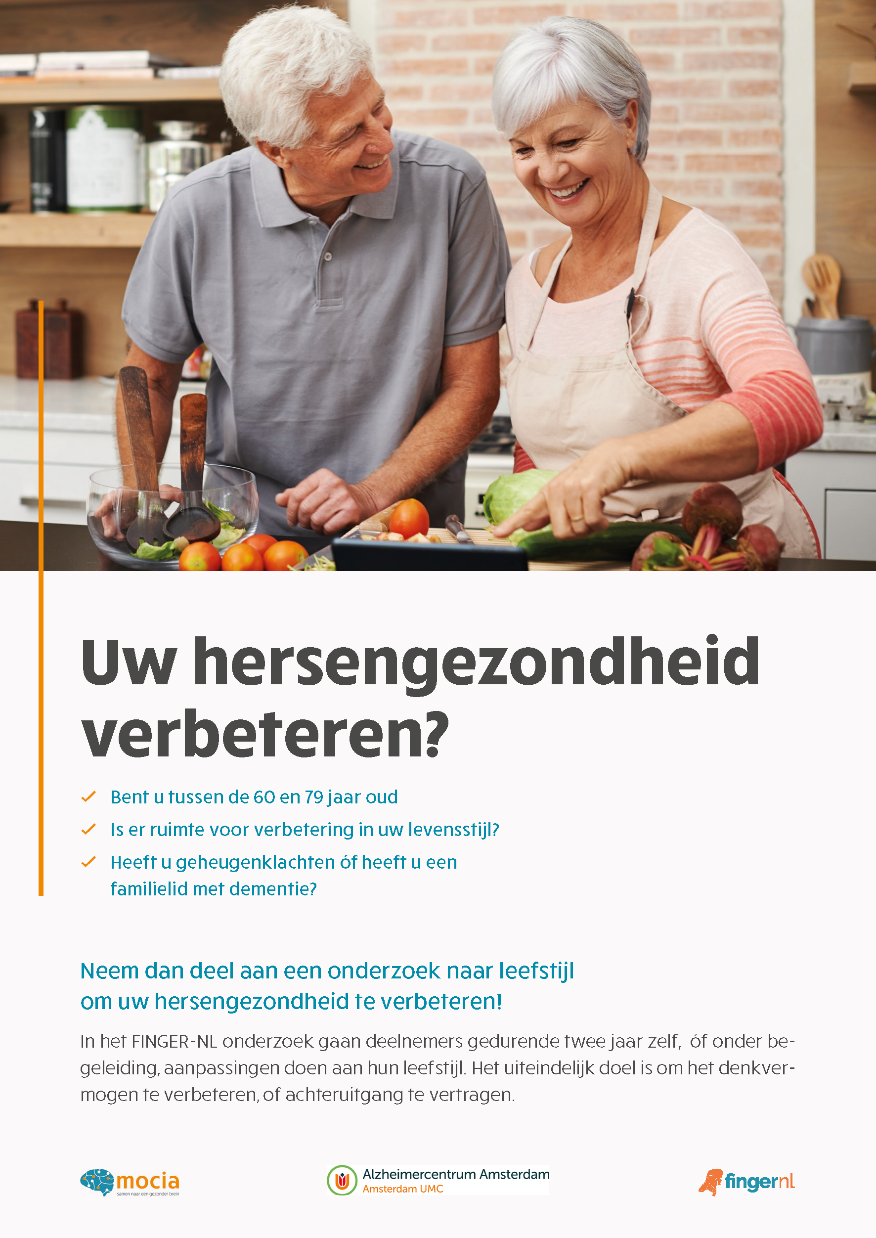
Supplementary Figure 2.** Example of promotional material designed by professional designer (https://verdickeme.nl/)

**Supplementary Table 2.** Number of new registrants recruited via different Facebook advertisement traced with UTM-code or self-reported through Facebook.

| Facebook | Content theme | Number of new registrants  (*n* = 4,678) |
| --- | --- | --- |
| A Blog | 1. Brain Health | 22 (1) |
|  | 1. Dementia | 5 (<1) |
| B Quiz | 1. Social engagement | 186 |
|  | 1. Sleep | 192 (4) |
| C Video with ambassador | 1. Dementia | 59 (1) |
|  | 1. Brain diseases | 48 (1) |
|  | 1. Lifestyle | 3,216 (69) |
| D Animation | 1. Prevention dementia | missing |
|  |  |  |
| Self-reported | - | 871 (18) |

Notes. *UTM* Urchin Tracking Module. Results are presented in frequency and percentages of total recruited via Facebook. Self-reported referral source and UTM were manually cross-checked for which UTM codes were leading. D1) data missing due to wrongly programmed UTM codes. See Supplementary Figure 1 for images.

**Supplementary Table 3**. Associations between the recruitment strategies (predictor) and sociodemographic and socioeconomic factors of under-represented groups.

| Recruitment (predictor) | Strategy 1)  Facebook advertisements | | Strategy 2)  Study launch on national television | | Strategy 3) Newspaper and online articles | | Strategy 4) Outreach via patient organizations | | Strategy 5)  Search engine  (eg. Google) | |  |  |
| --- | --- | --- | --- | --- | --- | --- | --- | --- | --- | --- | --- | --- |
| Under-represented groups | OR | 95%CI | OR | 95%CI | OR | 95%CI | OR | 95%CI | OR | 95%CI | *n* | R^2^_Tjur_ |
| Male sex | **0.60** | **0.53-0.68** | 1.16 | 1.01-1.33 | **1.40** | **1.23-1.59** | **0.62** | **0.51-0.76** | 0.83 | 0.68-1.02 | 12,750 | 0.026 |
| Migration background | 0.89 | 0.74-1.08 | 0.97 | 0.79-1.20 | **0.73** | **0.59-0.89** | 1.00 | 0.75-1.33 | **2.17** | **1.68-2.09** | 12,181 | 0.007 |
| Vocational education or less | **1.60** | **1.42-1.81** | **1.49** | **1.30-1.71** | **0.78** | **0.68-0.89** | 0.87 | 0.72-1.06 | 0.98 | 0.80-1.20 | 12,759 | 0.020 |
| Equivalent month income<€2415 | **1.35** | **1.20-1.52** | **1.55** | **1.35-1.77** | **0.71** | **0.62-0.80** | 1.02 | 0.85-1.22 | **1.43** | **1.17-1.74** | 12,117 | 0.022 |

Notes: *OR* odds ratio, *CI* confidence interval; significant outcomes (p < .05) are displayed in bold where odds ratio greater than one represents recruitment favouring under-represented groups. Associations are based on unadjusted univariate binomial logistic regression models where ‘Strategy 6) Other researcher outreach’ was set as the reference group and ‘Strategy 7) Other not specified/specific’ was excluded. For income, ‘Preferred not to say’ answers were imputed using a univariate imputation method with classification and regressions tree prediction based on age, sex and education. Other observations with missing data were excluded from the model.
